# Senolytic therapy alleviates physiological human brain aging and COVID-19 neuropathology

**DOI:** 10.1038/s43587-023-00519-6

**Published:** 2023-11-13

**Authors:** Julio Aguado, Alberto A. Amarilla, Atefeh Taherian Fard, Eduardo A. Albornoz, Alexander Tyshkovskiy, Marius Schwabenland, Harman K. Chaggar, Naphak Modhiran, Cecilia Gómez-Inclán, Ibrahim Javed, Alireza A. Baradar, Benjamin Liang, Lianli Peng, Malindrie Dharmaratne, Giovanni Pietrogrande, Pranesh Padmanabhan, Morgan E. Freney, Rhys Parry, Julian D. J. Sng, Ariel Isaacs, Alexander A. Khromykh, Guillermo Valenzuela Nieto, Alejandro Rojas-Fernandez, Thomas P. Davis, Marco Prinz, Bertram Bengsch, Vadim N. Gladyshev, Trent M. Woodruff, Jessica C. Mar, Daniel Watterson, Ernst J. Wolvetang

**Affiliations:** 1 Australian Institute for Bioengineering and Nanotechnology, University of Queensland, St Lucia, Queensland Australia; 2 School of Chemistry and Molecular Biosciences, University of Queensland, St Lucia, Queensland Australia; 3 School of Biomedical Sciences, Faculty of Medicine, University of Queensland, St Lucia, Queensland Australia; 4 Division of Genetics, Department of Medicine, Brigham and Women’s Hospital, Harvard Medical School, Boston, MA USA; 5 Belozersky Institute of Physico-Chemical Biology, Moscow State University, Moscow, Russia; 6 Institute of Neuropathology and Center for Basics in NeuroModulation, Faculty of Medicine, University of Freiburg, Freiburg, Germany; 7 Centre for Pharmaceutical Innovation, School of Pharmacy and Medical Sciences, UniSA Clinical and Health Sciences, The University of South Australia, Adelaide, South Australia Australia; 8 Clem Jones Centre for Ageing Dementia Research, Queensland Brain Institute, University of Queensland, Brisbane, Queensland Australia; 9 Australian Infectious Disease Research Centre, Global Virus Network Centre of Excellence, Brisbane, Queensland Australia; 10 Institute of Medicine, Faculty of Medicine & Center for Interdisciplinary Studies on the Nervous System, CISNE, Universidad Austral de Chile, Valdivia, Chile; 11 Nuffield Department of Medicine, University of Oxford, Oxford, UK; 12 Berking Biotechnology, Valdivia, Chile; 13 Signalling Research Centers BIOSS and CIBSS, University of Freiburg, Freiburg, Germany; 14 Faculty of Medicine, Clinic for Internal Medicine II, Gastroenterology, Hepatology, Endocrinology, and Infectious Disease, University Medical Center Freiburg, Freiburg, Germany; 15 Broad Institute of MIT and Harvard, Cambridge, MA USA

**Keywords:** Neural ageing, SARS-CoV-2, Senescence, Ageing

## Abstract

Aging is a major risk factor for neurodegenerative diseases, and coronavirus disease 2019 (COVID-19) is linked to severe neurological manifestations. Senescent cells contribute to brain aging, but the impact of virus-induced senescence on neuropathologies is unknown. Here we show that senescent cells accumulate in aged human brain organoids and that senolytics reduce age-related inflammation and rejuvenate transcriptomic aging clocks. In postmortem brains of patients with severe COVID-19 we observed increased senescent cell accumulation compared with age-matched controls. Exposure of human brain organoids to severe acute respiratory syndrome coronavirus 2 (SARS-CoV-2) induced cellular senescence, and transcriptomic analysis revealed a unique SARS-CoV-2 inflammatory signature. Senolytic treatment of infected brain organoids blocked viral replication and prevented senescence in distinct neuronal populations. In human-ACE2-overexpressing mice, senolytics improved COVID-19 clinical outcomes, promoted dopaminergic neuron survival and alleviated viral and proinflammatory gene expression. Collectively our results demonstrate an important role for cellular senescence in driving brain aging and SARS-CoV-2-induced neuropathology, and a therapeutic benefit of senolytic treatments.

## Abstract (web summary)

Aguado et al. show that SARS-CoV-2 induces senescence in human brain organoids and in the brains of COVID-19-infected mice and humans. They demonstrate the therapeutic potential of senolytic therapy in protection against COVID-19-induced brain aging.

## Main

Although SARS-CoV-2 is primarily a respiratory viral pathogen and the cause of COVID-19, persistent postacute infection syndromes (PAISs) derived from viral infections including SARS-CoV-2 are emerging as a frequent clinical picture^1,2^. In fact most patients with COVID-19, including individuals with or without comorbidities and even asymptomatic patients, often experience a range of neurological complications^3,4^. ‘Long-COVID’ is a type of PAIS that is gaining notable awareness, with patients reporting persistent manifestations such as hyposmia, hypogeusia, sleep disorders and substantial cognitive impairment, the latter affecting approximately one in four COVID-19 cases^5–7^. These clinical symptoms are supported by ample evidence of SARS-CoV-2 infectivity in multiple cell types of the nervous system^8–16^ and significant structural changes in the brains of patients with COVID-19 (ref. ^17^). Furthermore, patient transcriptomic data from postmortem brain tissue indicate associations between the cognitive decline observed in patients with severe COVID-19 and molecular signatures of brain aging^18^. In agreement with this observation, postmortem patient biopsies show that SARS-CoV-2-infected lungs accumulate markedly higher levels of senescence compared with uninfected counterparts^19^, a cellular phenotype known to contribute to both organismal aging^20^ and comorbidities such as chronic degenerative conditions^21^. Importantly, while recent data support a role for senescent cells in driving neurodegeneration and cognitive decline in both in vivo models of neuropathology^22,23^ and physiologically aged mice^24^, their contribution to COVID pathology in the central nervous system (CNS) and to human tissue brain aging remains unknown.

Over the past decade numerous strategies have been developed to target senescent cells^25^. Among these, the pharmacological removal of senescent cells with senolytic drugs has become one of the most explored interventions, with many currently in human clinical trials^26^. A group of these senolytics—such as the cocktail of dasatinib plus quercetin (D + Q) and fisetin—exhibit blood–brain barrier permeability following oral administration^22,27^, making these formulations particularly valuable for testing the contribution of senescence in the brain in vivo.

In the present study we first document the efficacy of multiple senolytic interventions in clearing senescent cells in physiologically aged human pluripotent stem cell (hPSC)-derived brain organoids (BOs). Transcriptomic analysis across individual senolytic treatments revealed a differential effect in modulating the senescence-associated secretory phenotype (SASP), with a distinctive impact of D + Q administration in rejuvenation of the BO transcriptomic aging clock. Importantly, we report an enrichment of senescent cells in postmortem brain tissue of patients with COVID-19 and further show a direct role for SARS-CoV-2 and highly neurotropic viruses such as Zika (ZIKV) and Japanese encephalitis (JEV) in evoking cellular senescence in human BOs. SARS-CoV-2 variant screening identified Delta (B.1.617.2) as the variant exerting the strongest induction of cellular senescence in human BOs, and spatial transcriptomic analysis of Delta-induced senescent cells unveiled a distinctive type of senescence that exhibits a transcriptional signature different from that of senescent cells that naturally emerge in in vitro aged uninfected BOs. Furthermore, senolytic treatment of SARS-CoV-2-infected BOs selectively removed senescent cells, lessened SASP-related inflammation and reduced SARS-CoV-2 RNA expression, indicating a putative role for senescent cells in facilitation of viral retention. Finally, to gain in vivo relevance of these findings, we examined the treatment effects of senolytic administration in transgenic mice expressing human angiotensin-converting enzyme 2 (hACE2)^8^ and previously infected with SARS-CoV-2. Our in vivo experiments demonstrate that senolytics improved clinical performance and survival, reduced viral load in the brain, improved survival of dopaminergic neurons, decreased astrogliosis and attenuated senescence and SASP gene expression in the brains of infected mice. Our findings therefore suggest a detrimental role for virus-induced senescence in acceleration of brain inflammation and the aging process in the CNS, and a potential therapeutic role for senolytics in the treatment of COVID-19 neuropathology.

## Results

### Senolytics target biological aging and senescence in physiologically aged human BOs

**Fig. 1:**
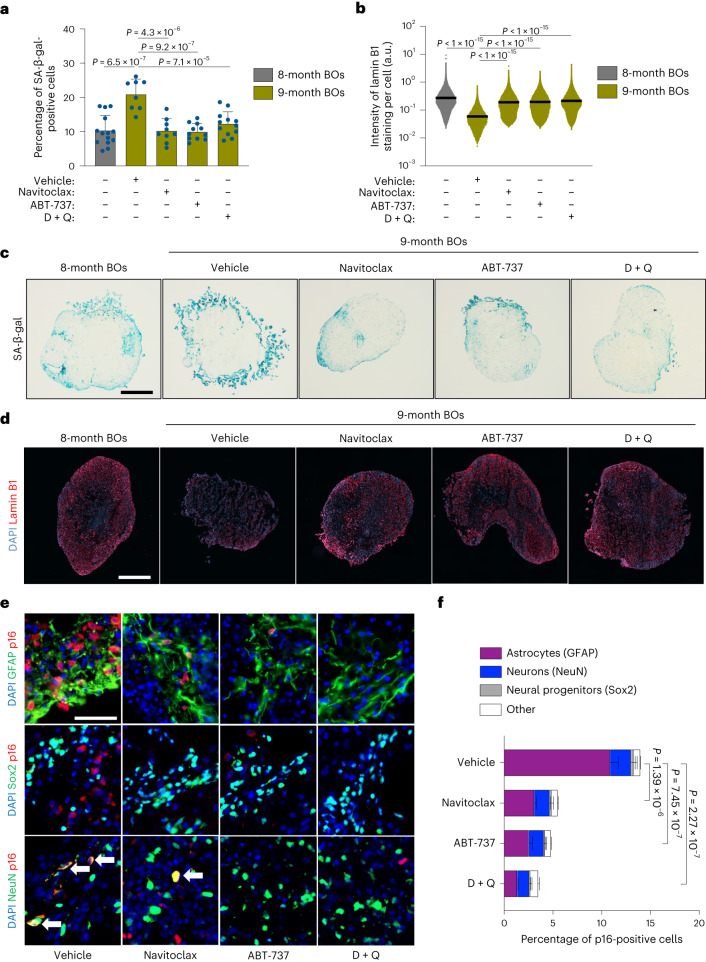
Long-term senolytic treatment prevents selective accumulation of senescent cells in physiologically aged human BOs. **a**–**f**, BOs were generated and grown in vitro for 8 months and subsequently exposed to two doses (one every 2 weeks) of either navitoclax (2.5 μM), ABT-737 (10 μM) or D + Q (D, 10 μM; Q, 25 μM) within the following month, after which organoids (*n* = 8–14) were collected for in situ analysis. **a**, SA-β-gal assay was performed on organoid sections. Each data point in the bar graph represents a single organoid analyzed. Data presented as mean ± s.d.; at least eight individual organoids were analyzed per condition; one-way analysis of variance (ANOVA) with Tukey’s multiple-comparison post hoc corrections. **b**, Lamin B1 staining was performed on organoid sections. Each data point in the scatter plot represents the integrated intensity of each cell within organoid sections. At least eight individual organoids were analyzed per condition; one-way ANOVA with Tukey’s multiple-comparison post hoc corrections. **c**,**d**, Representative images from quantifications shown in **a**,**b**, respectively. Scale bar, 0.3 mm. **e**, Representative immunofluorescent images of regions from organoids treated with the indicated senolytics and vehicle control. Samples were individually immunolabeled with antibodies against GFAP, Sox2 and NeuN and co-stained for p16. Arrows indicate coimmunoreactivity of NeuN and p16. Scale bar, 50 µm. **f**, Bar graphs showing colocalization quantification performed on organoid sections. Data presented as mean ± s.d.; three individual organoids were analyzed per condition; one-way ANOVA with Tukey’s multiple-comparison post hoc corrections. a.u., arbitrary units. Source data

**Extended Data Fig. 1:**
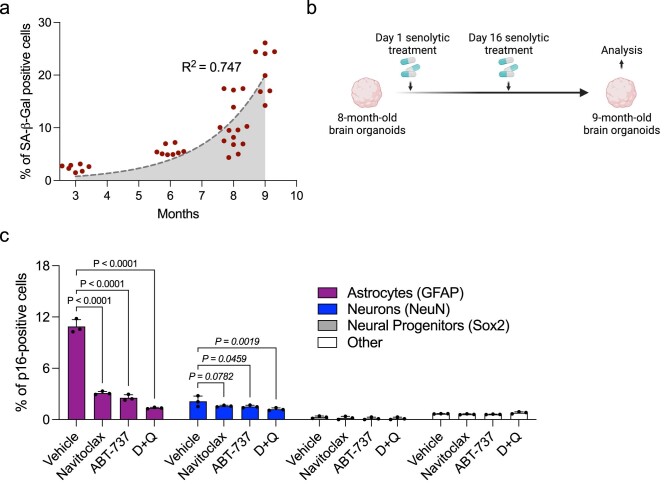
Characterization of cellular senescence in physiologically aged human BOs. **(a)** Scatter plot shows the number of SA-β-gal-positive cells per organoid. Each data point in the bar graph represents a single organoid analysed. Dotted lines represent the fitted non-linear regression curve. **(b)** Schematic representation of experimental design that applies to Figs. 1,2 and to Extended Data Fig. 1b-c. 8-month-old human BOs were exposed to two doses of the senolytic treatments navitoclax (2.5 μM), ABT-737 (10 μM) or D + Q (D: 10 μM; Q: 25 μM): the first one on day 1 and the second dose on day 16. Analysis was performed at the end time point of the 1-month experiment as well as at initial timepoint of 8 months organoid culture. **(c)** Bar graphs show the percentage of p16-positive cells co-localising with the indicated brain cell type markers. Each data point in the bar graph represents a single organoid analysed. Data are presented as mean values ± s.d.; 3 individual organoids were analysed per condition; one-way ANOVA with Dunnett’s multiple-comparison post-hoc corrections. Source data

To model the efficacy of senolytics in clearing senescent cells from human brain tissue models, we generated human BOs from embryonic stem cells. We next physiologically aged these for a period of 8 months because, at this timepoint, senescence accumulation reaches an exponential phase (Extended Data Fig. 1a) and therefore constitutes the optimal time-window for investigation of the impact of senolytics. At this stage we exposed BOs to two doses of senolytics for 1 month at 2-weekly intervals (Extended Data Fig. 1b). We tested the Bcl-2 inhibitors navitoclax and ABT-737, as well as the D + Q senolytic drug combination, and quantified the abundance of cells exhibiting senescence-associated β-galactosidase (SA-β-gal) activity. Exposure to senolytics resulted in significantly lower SA-β-gal activity compared with vehicle-treated controls (Fig. 1a,c), indicating that all treatments had eliminated a large number of senescent cells in treated BOs. In agreement with this, analysis of the expression of lamin B1 protein, a nuclear lamina marker often downregulated in senescence^28^, within organoid sections revealed a significantly higher content of lamin B1 in senolytic-treated organoids compared with control counterparts (Fig. 1b,d), further indicating that senolytics had cleared senescent cells by enrichment for lamin B1^High^ cell populations. We next interrogated the brain cell types responsible for senescence phenotypes by coimmunolabeling with p16 senescence marker. Notably we found that >75% of cells positive for p16 coimmunostained with astrocytes (glial fibrillary acidic protein (GFAP)-positive cells) whereas ~15% colocalized with mature neurons positive for NeuN antigen (Fig. 1e,f), with these populations of brain cells accounting for >90% of p16-positive cells. Importantly, senolytic interventions exerted a significant impact on reducing the population of senescent astrocytes, where the combination of D + Q exhibited the most potent effect (Fig. 1e,f and Extended Data Fig. 1c). The impact of senolytics on reduction of senescent neurons was less evident, where navitoclax failed to show a significant difference compared with vehicle-treated organoids, while D + Q exerted the most significant effect in alleviation of cellular senescence in neurons across all interventions tested (Extended Data Fig. 1c).

**Fig. 2:**
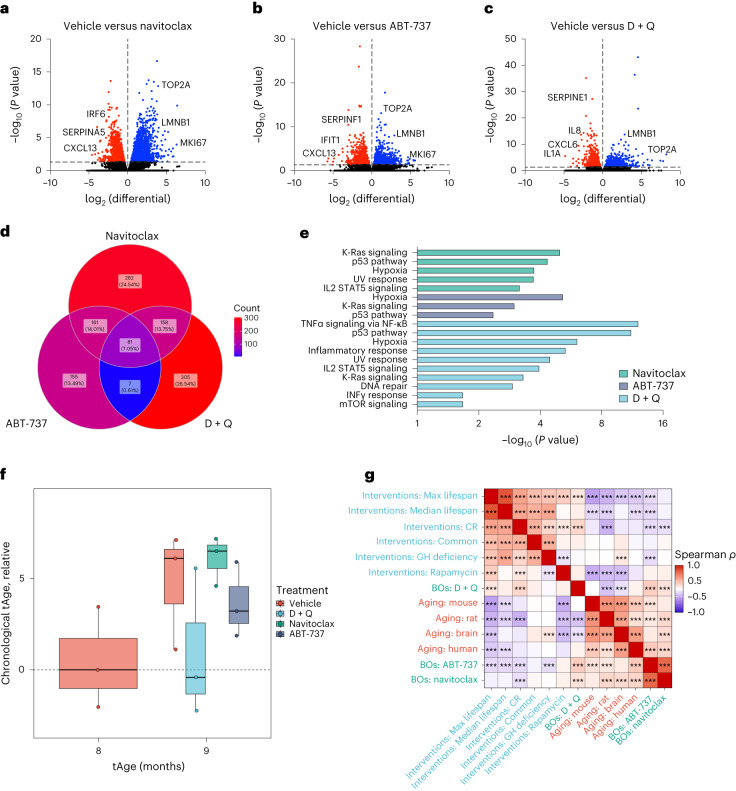
Transcriptomic characterization of distinct senolytic interventions on brain aging hallmarks. **a**–**g**, BOs were generated and grown in vitro for 8 months and subsequently exposed to two doses (one every 2 weeks) of either navitoclax (2.5 μM), ABT-737 (10 μM) or D + Q (D, 10 μM; Q, 25 μM) within the following month, after which organoids were collected and subjected to bulk RNA-seq analysis. **a**–**c**, Volcano plots showing vehicle-treated versus navitoclax- (**a**), ABT-737- (**b**) and D + Q-treated (**c**) BO differential expression of upregulated (blue) and downregulated (red) mRNAs (*P* < 0.05, log_2_FC > 0). **d**, Venn diagram showing differentially repressed senescence-associated mRNAs among senolytic-treated organoids, defined by significance *P* < 0.05 and log_2_FC > 0. **e**, GSEA was carried out using aging hallmark gene sets from the Molecular Signature Database. Statistically significant signatures were selected (*P* < 0.05, false discovery rate < 0.25) and placed in order of NES. Bars indicate pathways enriched in individual senolytic treatments compared with vehicle-treated BOs. **f**, Transcriptomic age (tAge) of organoids treated with either vehicle or senolytic compounds assessed using the brain multispecies aging clock. Three individual organoids were analyzed per condition. Box-and-whisker plot (minimum, 25th percentile; median, 75th percentile; maximum). **g**, Spearman correlation between gene expression changes induced by senolytics in aged organoids and signatures of aging and established lifespan-extending interventions based on functional enrichment output. NES calculated with GSEA were used to evaluate correlations between pairs of signatures. Source data

**Extended Data Fig. 2:**
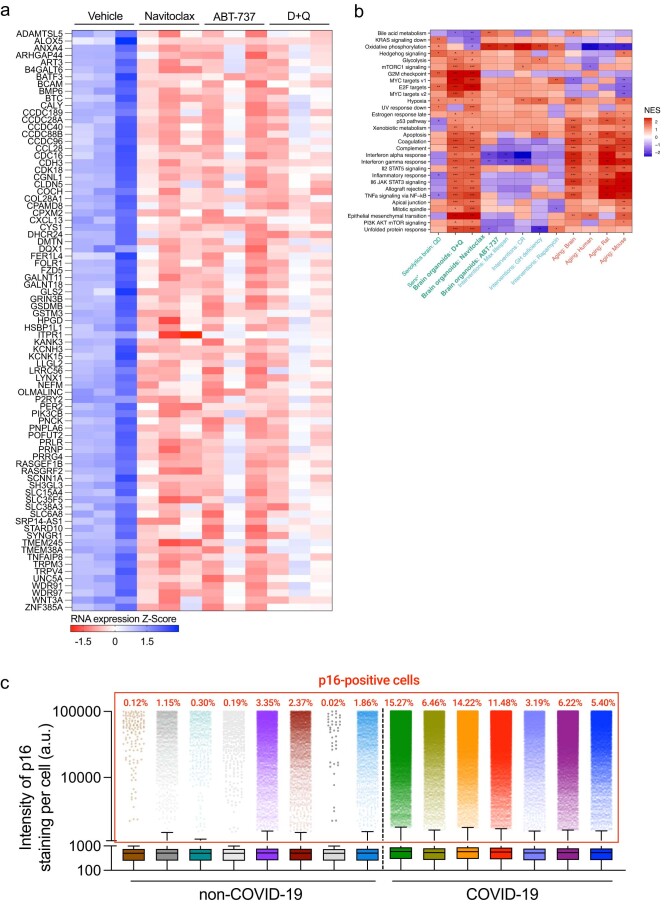
Senolytic-driven transcriptomic changes in BOs and senescence single-cell analysis of postmortem human brain frontal cortex. **(a)** Heat map shows senescence-associated RNA transcriptomic expression of downregulated mRNAs shared across all three senolytic interventions. **(b)** Functional enrichment analyses of gene expression signatures and multiple senolytic treatment of BOs. Heat map cells are coloured based on the normalized enrichment score (NES). **(c)** Box-and-whisker plots (Tukey’s minimum, 25th percentile, median, 75th percentile, Tukey’s maximum) shows the intensity in normalised arbitrary units (a.u.) of p16 protein. Each dot represents outlier cells of a total of 2,794,379 individual brain cells across 7 COVID-19 and 8 non-COVID-19 patients. p16-positive cells were assigned when a cell’s normalised intensity surpassed the value of 1,000. Source data

We next performed whole-organoid RNA sequencing (RNA-seq) to compare the transcriptomes of senolytic-treated and vehicle control 9-month-old BOs. Consistent with our protein expression data (Fig. 1b,d), lamin B1 messenger RNA levels were significantly upregulated in all three senolytic-treated organoids compared with vehicle-treated counterparts (Fig. 2a–c). We further identified 81 senescence-associated messenger RNAs (including proinflammatory genes *CXCL13* and *TNFAIP8*) that were consistently suppressed following all three senolytic interventions (Fig. 2d and Extended Data Fig. 2a). We also noticed, however, that individual senolytic treatments exerted substantially different effects in modulation of SASP and other senescence-associated mRNAs (Fig. 2a–c). For instance, SERPINF1 mRNA level was significantly repressed following ABT-737 administration (Fig. 2b) while D + Q did not modulate *SERPINF1* gene expression but markedly supressed IL8, SERPINE1 and IL1A mRNA levels (Fig. 2c). Compared with navitoclax and ABT-737—compounds that modulate the expression of multiple shared genes enriched for a few pathways (for example, K-Ras signaling; Fig. 2e)—D + Q had a more broad-spectrum effect, mitigating multiple proinflammatory pathways characteristic of cellular senescence including NF-κB and IFNγ signaling (Fig. 2e and Extended Data Fig. 2b). In addition, we identified mTOR as a significantly supressed pathway following D + Q treatment (Fig. 2e), validating the effects reported for Q as an inhibitor of mTOR kinase. We next performed aging clock predictions based on whole-transcriptome sequencing to further explore the impact of senolytics on the aging process. Remarkably, in addition to their senolytic mechanisms of action, D + Q treatments on 9-month-old organoids reverted their gene expression age to levels comparable to 8-month-old counterparts according to transcriptomic brain aging clock analysis (Fig. 2f), a phenotype not recapitulated by the other two senolytics tested. Besides negative association with known signatures of aging^29^ (Fig. 2g), gene expression changes induced by D + Q treatment were positively correlated with mammalian signatures of established lifespan-extending interventions^30^, such as caloric restriction (CR) and rapamycin administration (Fig. 2g), indicating a health-promoting role of D + Q in targeting cellular senescence and biological aging in human CNS tissues.

### SARS-CoV-2 infection triggers senescence in brains of patients with COVID-19 and in human BOs

**Fig. 3:**
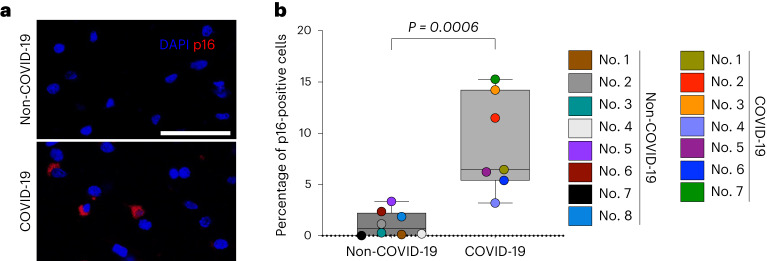
Brains of patients with COVID-19 exhibit increased accumulation of p16 senescent cells. **a**, Immunofluorescence images showing DAPI (blue) and p16 (red) immunoreactivity in sections of frontal cortex regions from patients with severe COVID-19 and age-matched non-COVID-related controls. Scale bar, 50 μm. **b**, Box-and-whisker plots (minimum, 25th percentile; median, 75th percentile; maximum) showing percentage of p16-positive cells. Each data point represents a single patient analyzed, with a total of 2,794,379 individual brain cells across seven patients with COVID-19 and eight non-COVID-19. Two-tailed Student’s *t*-test. Source data

Given the observed neuroinflammatory effects of SARS-CoV-2 infection during acute COVID-19 disease^31^ and its association with molecular signatures of aging in patient brains^18^, we postulated that part of this proinflammatory aging-promoting environment is brought about by SARS-CoV-2-induced senescence in the brain. To test this hypothesis we quantified the prevalence of senescent cells in postmortem frontal cortex from age-matched brains of patients that either died following severe COVID-19 or that died from noninfectious and non-neurological causes. Notably, in situ high-throughput analysis of >2.7 million single cells across 15 individual brain samples (seven COVID-19 and eight non-COVID-19 frontal cortex sections) revealed increased p16 immunoreactivity frequencies in the brains of patients with COVID-19, with an increase of over sevenfold in the number of p16-positive cells compared with non-COVID-19 age-matched controls (Fig. 3 and Extended Data Fig. 2c). These results suggest a potential role for SARS-CoV-2 in triggering cellular senescence, a cellular phenotype that contributes to cognitive decline and that could pose a risk regarding the acceleration of neurodegenerative processes associated with long-COVID.

**Fig. 4:**
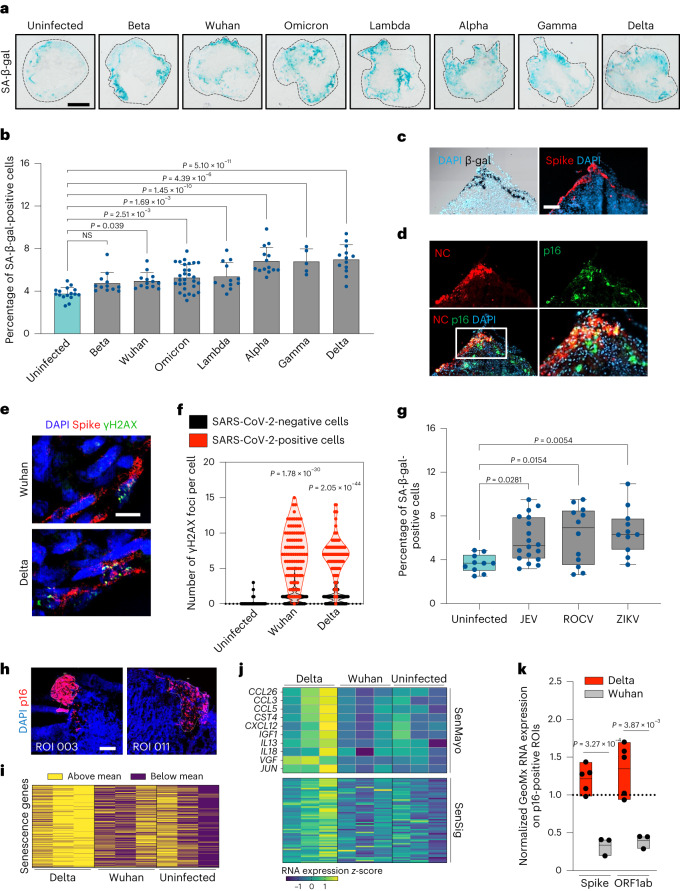
Neurotropic viral infections elicit virus-induced senescence in human BOs. **a**, SARS-CoV-2 variant screening was performed on BOs and monitored for SA-β-gal activity at 5 dpi. Scale bar, 0.3 mm. **b**, Quantification of data presented in **a**. Bar graphs show the percentage of SA-β-gal-positive cells. Each data point in the bar graph represents a single organoid analyzed (*n* = 5–29). Data presented as mean ± s.d.; one-way ANOVA with Dunnett’s multiple-comparison post hoc corrections. **c**, Representative images of Delta-infected organoid sections stained for SA-β-gal and SARS-CoV-2 Spike protein. **d**, Representative images of the region shown in **c** coimmunolabeled for p16 and SARS-CoV-2 nucleocapsid (NC). **c**,**d**, One representative experiment out of two is shown. Scale bar, 100 µm. **e**, Organoids infected for 5 days with SARS-CoV-2 variants were stained for γH2AX and SARS-CoV-2 spike protein. Scale bar, 40 μm. **f**, Quantification of data presented in **e**. Scatter plot showing the number of γH2AX foci per cell in infected regions (red) versus uninfected counterparts (black). Each data point in the scatter plot represents a single cell analyzed; at least 400 cells per condition were analyzed; *n* = 3 BOs; two-tailed Student’s *t*-test. **g**, Human BOs were infected with JEV, ROCV and ZIKV and monitored for SA-β-gal activity at 5 dpi. Box plots show percentage of SA-β-gal-positive cells. Each data point represents a single organoid (*n* = 10–18) analyzed. Box-and-whisker plots (minimum, 25th percentile; median, 75th percentile; maximum); one-way ANOVA with multiple-comparison post hoc corrections. **h**–**k**, Uninfected, Wuhan- and Delta-infected human BOs were subjected to ROI selection based on p16 protein expression for spatial profiling by the Nanostring GeoMX digital spatial profiler assay, and further sequenced for the GeoMx Human Whole Transcriptome Atlas. **h**, Representative p16-positive ROIs. Scale bar, 200 µm. **i**, Heatmap of polarity showing expression above (blue) and below (red) the mean for each differentially heightened SASP mRNA of Delta-infected, p16-positive ROIs. **j**, SenMayo and SenSig senescence signature heatmap gene expression of Delta-infected p16-positive cells. **k**, Floating bars (minimum, mean, maximum) showing expression enrichment of SARS-CoV-2 RNAs (Spike, ORF1ab) for each SARS-CoV-2 variant. Each data point in the box plot represents a normalized FC value of SARS-CoV-2 RNAs of p16-positive ROIs relative to p16-negative counterparts (indicated by grid line). *n* = 3–5 p16-positive ROIs were analyzed per condition; two-tailed Student’s *t*-test. NS, not significant. Source data

**Extended Data Fig. 3:**
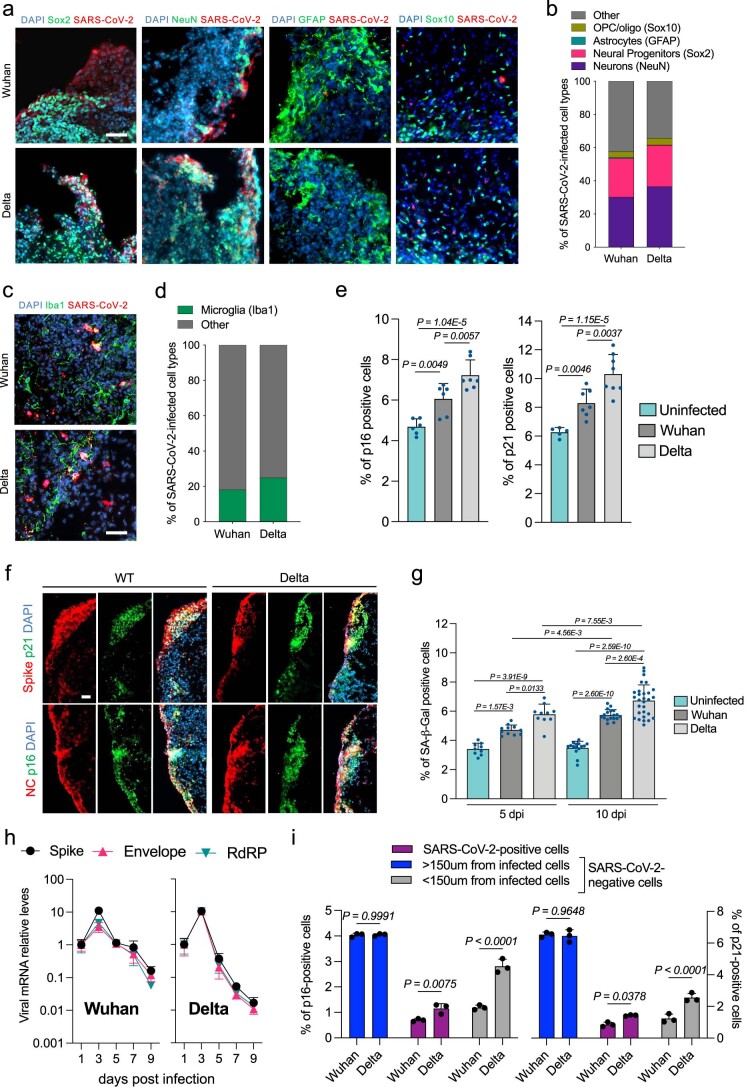
Characterization of neurotropic SARS-CoV-2-induced senescence. **(a)** Representative images of neural progenitors (Sox2), neurons (NeuN), or astrocytes (GFAP) co-stained with SARS-CoV-2 nucleocapsid protein. Human BOs were 3 month-old at time of infection with the indicated SARS-CoV-2 variants at MOI 1 and collected at 5dpi. Scale bar, 50 µm. One representative experiment out of two is shown. **(b)** Stacked bar graphs show quantifications from **a**. **(c,d)** Microglia-containing organoids were generated as previously described^56^, these organoids were matured for 3 months prior to SARS-CoV-2 infection at MOI 1 and collected at 5dpi. **(c)** Representative images of microglia (Iba1) co-stained with SARS-CoV-2 nucleocapsid protein. Scale bar, 50 µm. **(d)** Stacked bar graphs show quantifications from **c**. **(e)** Bar graphs show the percentage of p16- and p21-positive cells. Each data point in the bar graph represents a single organoid (n = 5-8) analysed at 5dpi. Data are presented as mean values ± s.d.; one-way ANOVA with Dunnett’s multiple-comparison post-hoc corrections. **(f)** Representative images of p16 and p21 co-stained with SARS-CoV-2 nucleocapsid and spike proteins. Scale bar, 50 µm. **(g)** Bar graphs show the percentage of SA-β-gal-positive cells. Each data point in the bar graph represents a single organoid (n = 9-29) analysed at 5 and 10dpi. Data are presented as mean values ± s.d.; one-way ANOVA with Dunnett’s multiple-comparison post-hoc corrections. **(h)** Total RNA from individual organoids infected with the indicated SARS-CoV-2 variants was used to quantify the indicated levels of viral RNAs and normalized to RPLP0 mRNA and compared to infected organoids at 1dpi. Error bars represent s.e.m.; n = 3 independent organoids; one-way ANOVA with multiple-comparison post-hoc corrections. **(i)** Bar graphs show the percentage of p16- and p21-positive cells within SARS-CoV-infected cells, and distal ( > 150 µm) and proximal ( < 150 µm) uninfected cells to SARS-CoV-2 infected cells positive for p16. Each data point in the bar graph represents a single organoid analysed. Data are presented as mean values ± s.d.; 3 individual organoids were analysed per condition; two-way ANOVA with multiple-comparison post-hoc corrections. Source data

To study the role of neurotropic viruses in aging-driven neuropathology, we exposed human BOs to different viral pathogens including SARS-CoV-2. Consistent with previous reports^8,9,12,16,32^, SARS-CoV-2 BO infections were detected largely within populations of neurons, neural progenitors and microglia (Extended Data Fig. 3a–d). To test putative virus-induced senescence phenotypes we screened seven SARS-CoV-2 variants by infecting human BOs at identical multiplicity of infection (MOI) and ranked them based on SA-β-gal activity as initial readouts of cellular senescence. Notably, most variants elicited a significant increase in SA-β-gal, with Delta (B.1.617.2) showing the strongest induction (Fig. 4a,b), and this was accompanied by an overall increase in the number of p16- and p21-positive cells (Extended Data Fig. 3e,f). In addition, serial sectioning of Delta-infected organoids revealed a distinctive colocalization between SA-β-gal and viral Spike protein (Fig. 4c), further supporting a role for SARS-CoV-2 in driving virus-induced senescence in the brain. This phenotype was confirmed when organoid sections were coimmunolabeled with antibodies against p16 and SARS-CoV-2 nucleocapsid antigens (Fig. 4d). Importantly, we observed a statistically greater senescence induction between BOs infected for 5 and 10 days (days post infection, dpi; Extended Data Fig. 3g). This occurred in the absence of viral replication, which was detectable only in the first 3 days (Extended Data Fig. 3h). Collectively these observations suggest that the increased senescence observed at 10 versus 5 dpi may have been the result of secondary senescence triggered by the initial SARS-CoV-2 infection and ensuing induced senescence phenotype. Indeed, in Delta-infected BOs, when monitoring peripheral senescence in close vicinity (<150 μm) to virus-infected senescent cells we observed an enrichment of senescent cells negative for SARS-CoV-2 (Extended Data Fig. 3i), further indicating putative bystander effects of SARS-CoV-2-infected cells in inducing secondary senescence at proximal sites of infection. Because of the mechanistic role of DNA damage in affecting most aging hallmarks^33^, including the onset of cellular senescence^34^, we next explored whether SARS-CoV-2 infection would lead to DNA double-strand break accumulation. Consistent with previous evidence^35^, we detected significantly heightened levels of phosphorylated histone H2AX at serine 139 (known as γH2AX) in SARS-CoV-2-infected organoid regions compared with uninfected organoid cells (Fig. 4e,f), indicating increased DNA damage response marks following SARS-CoV-2 infection. Importantly, virus-induced senescence also became detectable in response to a variety of human neurotropic viruses, including JEV, Rocio virus (ROCV) and ZIKV in human BOs (Fig. 4g).

**Extended Data Fig. 4:**
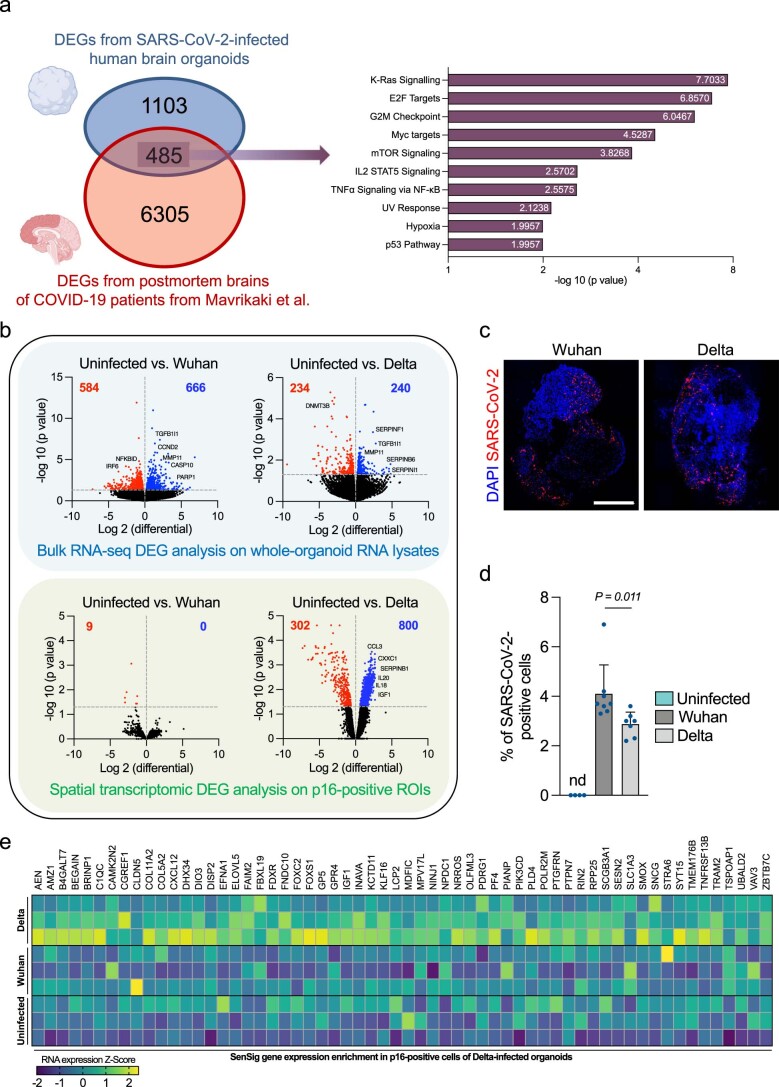
Transcriptional characterization of virus-induced senescence. **(a)** Venn diagram on the left shows 485 differentially expressed genes shared across SARS-CoV-2-infected organoids and postmortem brains of COVID-19 patients defined with a significance adjusted P < 0.05 and log_2_FC > 0. On the right panel, bar graph indicates the pathways enriched within this 485-gene cohort. Gene Set Enrichment Analysis was carried out using aging hallmark gene sets from the Molecular Signature Database. The statistically significant signatures were selected (P < 0.05, FDR < 0.25). **(b)** Volcano plots show uninfected versus either Wuhan or Delta-infected brain organoid differential expression of upregulated (blue) and downregulated (red) RNAs (P < 0.05, log_2_FC > 0). DEG analysis was performed from whole-organoid RNA-seq data and p16-positive senescent-cell regions of interest (ROIs) from NanoString spatial transcriptomic sequencing. **(c)** Representative images of SARS-CoV-2 nucleocapsid protein immunoreactivity on BOs infected with the indicated SARS-CoV-2 variants and analysed at 5 days post infection. Scale bar, 500 µm. **(d)** Bar graph shows quantifications of nucleocapsid-positive cells from BOs (n = 4-8) uninfected and infected with the indicated SARS-CoV-2 variants and analysed at 5 dpi. Each data point in the bar graph represents a single organoid analysed. Data are presented as mean values ± s.d.; one-way ANOVA with multiple-comparison post-hoc corrections. **(e)** SenSig senescence signature heat map gene expression of Delta-infected p16-positive cells. Source data

Because SARS-CoV-2 infection is coupled with cognitive decline and signatures of aging, we further assessed associations of transcriptomic changes in patients with COVID-19 and SARS-CoV-2-infected human BOs. Specifically, we compared postmortem frontal cortex transcriptomic data from a COVID-19 cohort study of 44 individual patient brains^18^ with bulk RNA-seq that we performed on human cortical BOs 10 dpi. Notably, among 1,588 differentially expressed genes (DEGs) between SARS-CoV-2-infected human BOs and uninfected counterparts, 485 genes (30.54%) were also differentially expressed in brain samples from patients with COVID-19. Of note, this common gene set was enriched for known aging and senescence pathways, identified in the hallmark gene set collection of the Molecular Signatures Database^36^ (Extended Data Fig. 4a).

**Extended Data Fig. 5:**
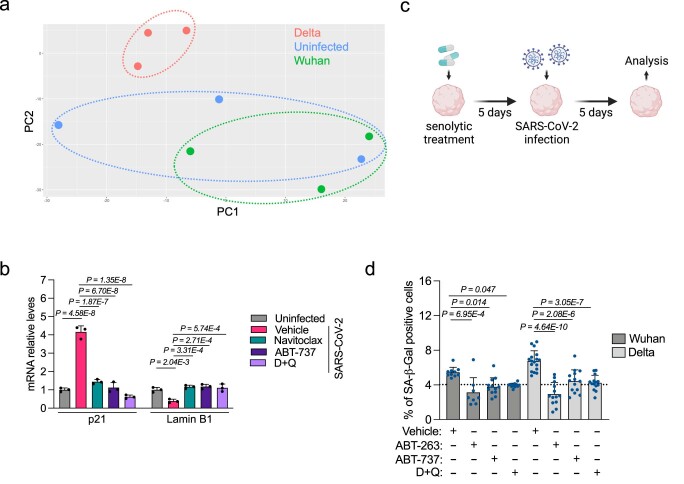
Effects of senolytic administration prior to SARS-CoV-2 infection. **(a)** Principal component analysis from NanoString spatial transcriptomic sequencing of p16-positive cells in the subspace defined by these differentially-expressed genes showing clustering of uninfected and Wuhan-infected human BOs away from the Delta-infected counterparts. **(b)** Total RNA from individual organoids uninfected or infected with the SARS-CoV-2 Delta variant was used to quantify Lamin B1 and p21 mRNA expression levels and normalized to RPLP0 mRNA and compared to infected vehicle controls. Data are presented as mean values ± s.d.; n = 3 independent organoids; one-way ANOVA with multiple-comparison post-hoc corrections. **(c)** Schematic representation of experimental design that applies to **d**. Human BOs were exposed to the indicated senolytic treatments for 5 days and subsequently SARS-CoV-2-infected at multiplicity of infection 1 for 5 additional days. Analysis was performed at the end time point of the 10-day experiment. **(d)** SA-β-gal activity was evaluated at 10 days post infection. Bar graphs show the percentage of SA-β-gal-positive cells. Each data point in the bar graph represents a single organoid (n = 6-17) analysed. Data are presented as mean values ± s.d.; one-way ANOVA with multiple-comparison post-hoc corrections. Source data

To better understand the differential effects of the ancestral Wuhan virus and Delta (B.1.617.2) SARS-CoV-2 variants on senescence induction in BOs, we next performed NanoString GeoMx spatial transcriptomic sequencing on p16 protein-expressing regions of interest (ROIs) within organoid sections (Fig. 4h). ROI selection was performed to enable the capture of targeted transcriptome from sufficient senescent cell tissue (>300 cells per ROI) to generate robust count data. Our bulk RNA-seq analysis revealed 1,250 DEGs in Wuhan-infected BOs compared with only 474 DEGs in Delta-infected counterparts (Extended Data Fig. 4b), a result possibly explained by the higher infectivity rate observed in Wuhan-infected organoids (Extended Data Fig. 4c,d). Strikingly, spatial transcriptome analysis of p16-positive cells identified >1,100 DEGs in Delta-infected organoids, an effect 100-fold greater than in Wuhan where only nine DEGs were detected (Extended Data Fig. 4b). This was explained by principal component analysis, where gene set space determined that Delta-infected ROIs were separable from overlapping transcriptomes from Wuhan-infected and uninfected senescent cell regions (Extended Data Fig. 5a). Following extensive analysis of significantly modulated gene expression in p16-positive ROIs of Delta-infected organoids, we identified 458 genes associated with cellular senescence that differentially clustered from Wuhan-infected and uninfected ROIs (Fig. 4i), and revealed a distinct enrichment of senescence gene sets SenMayo^37^ and SenSig^38^ within Delta-infected ROIs (Fig. 4j and Extended Data Fig. 4e). Importantly, this unique Delta-specific senescence transcriptional signature was detected in the presence of heightened normalized SARS-CoV-2 gene expression in Delta compared to p16-positive cells of Wuhan-infected organoids (Fig. 4k).

Taken together, these results demonstrate a direct role for SARS-CoV-2 and neurotropic flaviviruses in fueling virus-induced senescence, and reveal a specific effect of Delta (B.1.617.2) in promoting the selective induction of a de novo transcriptional signature and simultaneous accumulation of SARS-CoV-2 in senescent cells of human BOs.

### Senolytics reduce SARS-CoV-2 viral expression and virus-induced senescence in human BOs

**Fig. 5:**
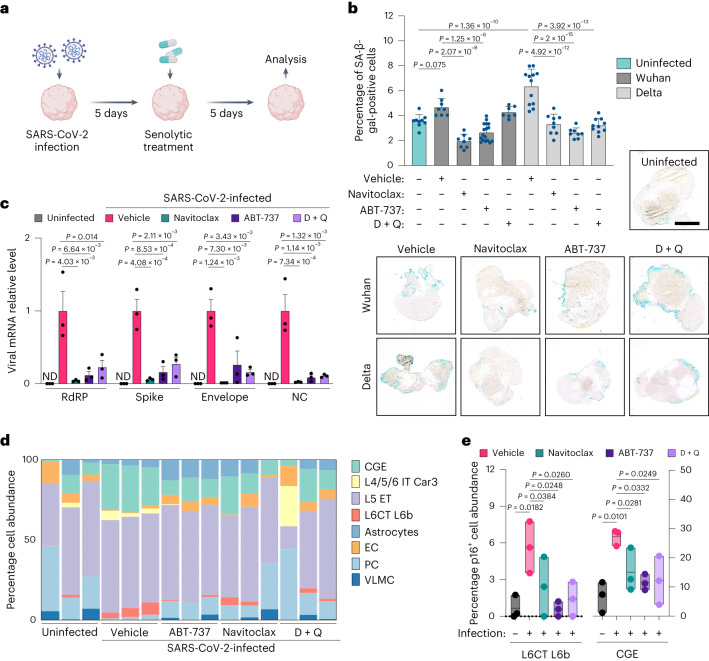
Senolytics clear virus-induced senescence in specific neuronal subtypes. **a**, Schematic representation of experimental design pertaining to **b**–**e**. Human BOs were SARS-CoV-2 infected at MOI 1 for 5 days and subsequently exposed to the indicated senolytic treatments for five additional days. Analysis was performed at the end timepoint of the 10-day experiment. **b**, SA-β-gal activity was evaluated at 10 dpi. Bar graphs showing percentage of SA-β-gal-positive cells. Each data point in the bar graph represents a single organoid (*n* = 7–17) analyzed. Data presented as mean ± s.d.; one-way ANOVA with multiple-comparison post hoc corrections. Scale bar, 0.3 mm. **c**, Total RNA from individual organoids uninfected or infected with the SARS-CoV-2 Delta variant was used to quantify the indicated levels of viral RNAs, normalized to *RPLP0* mRNA and compared with infected vehicle controls. Error bars represent s.e.m.; *n* = 3 independent organoids; one-way ANOVA with multiple-comparison post hoc corrections; ND, not detected; RdRP, RNA-dependent RNA polymerase. **d**, Stacked bars showing NanoString GeoMx deconvolved p16-positive ROI cell abundance using constrained log-normal regression from organoids either uninfected or infected with the SARS-CoV-2 Delta variant. L4/5/6 IT Car3, glutamatergic neurons; L5 ET, cortical layer 5 pyramidal neurons; L6CT L6b, corticothalamic (CT) pyramidal neurons in layer 6; CGE, GABAergic ganglionic eminence neurons; VLMC, vascular and leptomeningeal cells. **e**, Floating bar graphs (minimum, mean, maximum) showing percentage of deconvolved p16-positive neuronal populations significantly modulated following SARS-CoV-2 Delta variant infection and subsequent senolytic interventions. *n* = 3 independent ROIs per condition tested; *P < 0.05, one-way ANOVA with multiple-comparison post hoc corrections. Source data

The results described so far support a functional role of SARS-CoV-2 in inducing brain cellular senescence. To investigate whether this virus-induced phenotype could be pharmacologically targeted, we next tested the impact of the selective removal of senescent cells with the same senolytic interventions that we previously showed as being effective in elimination of senescent cells from physiologically aged organoids (Fig. 5a). We observed that senolytic treatments 5 days post SARS-CoV-2 infection significantly reduced the number of organoid cells showing SA-β-gal activity (Fig. 5b). Notably, senolytic treatment of Delta-infected BOs had an overall more prominent and statistically significant effect on reduction of cellular senescence compared with Wuhan-infected counterparts, consistent with the stronger virus-induced senescence phenotype observed following Delta infections in our initial SARS-CoV-2 variant screening (Fig. 4a,b). Moreover, senolytics were able to revert p21 upregulation and lamin B1 loss induced by Delta infections (Extended Data Fig. 5b). Remarkably, treatment with senolytics reduced viral load in BOs up to 40-fold as measured by intracellular SARS-CoV-2 RNA levels (Fig. 5c), indicating a putative role for senescent cells as reservoirs that may preferentially facilitate viral replication. Notably, pretreatment of BOs with senolytics before SARS-CoV-2 infection significantly reduced virus-induced senescence, suggesting that naturally emerging senescent cells contribute to BO viral entry and the subsequent onset and spread of virus-induced senescence (Extended Data Fig. 5c,d). To characterize the cell type specificity of SARS-CoV-2-induced senescence we performed deconvolution of spatial GeoMx transcriptomic data from p16-positive cells (Fig. 5d), a type of analysis we use to enable cell abundance estimates from gene expression patterns based on a training matrix of single-cell sequencing data from the Allen Human Brain Atlas^39^. This identified layer 6 corticothalamic neurons (L6CT L6b, over ninefold induction) and GABAergic ganglionic eminence neurons (CGE, over fourfold induction) as the two neuronal populations that showed significantly increased senescence incidence following SARS-CoV-2 infections in BOs (Fig. 5e)—two brain cell populations that are vital in modulation of neural circuitry and processing of incoming sensory information^40^. Importantly, all three senolytic treatments tested prevented the accumulation of cellular senescence in both L6CT L6b and CGE BO cell populations (Fig. 5e).

### Senolytic treatments mitigate COVID-19 brain pathology in vivo

**Fig. 6:**
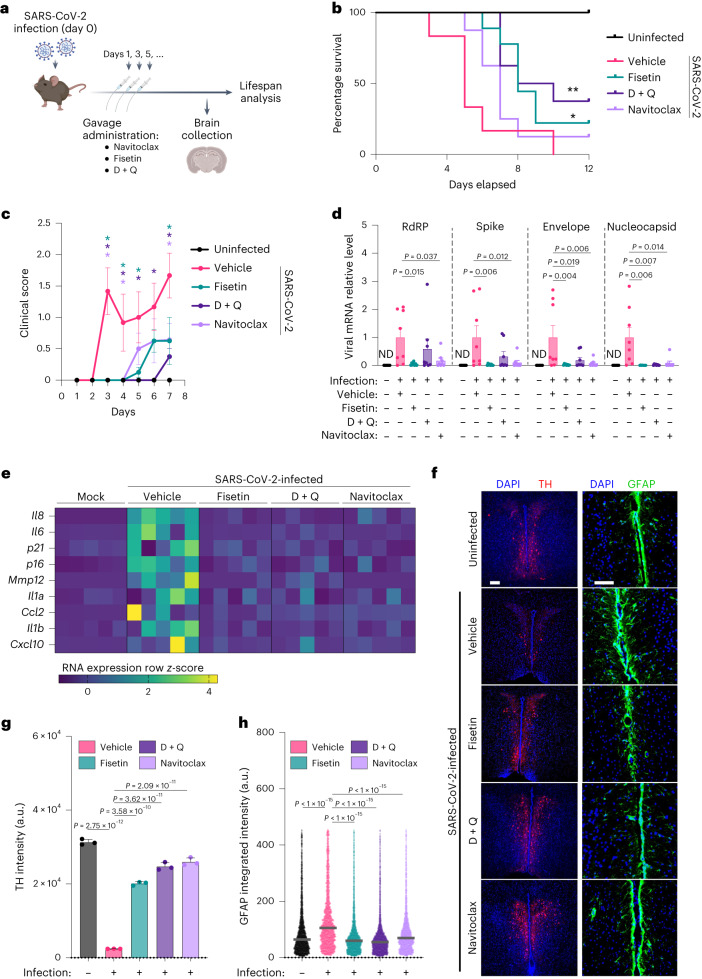
Senolytic treatments mitigate COVID-19 brain pathology in vivo. **a**, Schematic representation of experimental design pertaining to **b**–**h**. K18-hACE2 transgenic mice were exposed to Delta variant infection on day 0 and subsequently treated with senolytics every other day starting on day 1. Mice were euthanized on day 5 for brain tissue characterization, as well as for end timepoint experiments to monitor clinical score and survival. **b**, Kaplan–Meier curve of uninfected mice (*n* = 3) and of infected mice treated with vehicle (*n* = 6), fisetin (*n* = 9), D + Q (*n* = 8) or navitoclax (*n* = 8). *P = 0.032 for vehicle versus fisetin curve comparison; **P = 0.0087 for vehicle versus D + Q curve comparison; log-rank (Mantel–Cox) test. **c**, Average combined behavioral and physical clinical score, over time, of uninfected mice (*n* = 3) and of SARS-CoV-2-infected mice treated with vehicle (*n* = 6), fisetin (*n* = 8), D + Q (*n* = 8) or navitoclax (*n* = 8). Error bars represent s.e.m.; color-coded *P < 0.05 for comparisons between vehicle and each color-coded senolytic treatment; one-way ANOVA with multiple-comparison post hoc corrections for every timepoint tested. **d**, Total RNA of individual brains from mice—either uninfected or Delta variant-infected and treated with senolytics—was used to quantify the indicated levels of viral RNAs and was normalized to *Rplp0* mRNA and compared with infected vehicle controls. Error bars represent s.e.m.; *n* = 8 mouse brains per condition; one-way ANOVA with multiple-comparison post hoc corrections. **e**, Total RNA of individual brains from mice— either uninfected or infected with SARS-CoV-2 Delta variant and treated with various senolytic interventions—was used to quantify mRNA expression levels of the indicated senescence and SASP RNAs and was normalized to *Rplp0* mRNA. Each column in the heatmap represents an individual mouse brain analyzed. **f**, Representative immunofluorescent images of brainstem sections from mice either uninfected or infected with the SARS-CoV-2 Delta variant and treated with the indicated senolytics. Samples were immunolabeled with antibodies against TH (red; scale bar, 100 µm) and GFAP (green; scale bar, 50 µm). **g**, Quantification of TH data presented in **f**. Bar graph showing the intensity of TH staining. Each data point in the bar graph represents average TH intensity analyzed per mouse brain (*n* = 3). Data presented as mean ± s.d.; ****P < 0.0001, one-way ANOVA with multiple-comparison post hoc corrections. **h**, Quantification of GFAP data presented in **f**. Dot plot shows the intensity of GFAP per cell. Each data point in the dot blot represents a single cell analyzed. ****P < 0.0001; three brains per condition were analyzed; one-way ANOVA with multiple-comparison post hoc corrections. Source data

**Extended Data Fig. 6:**
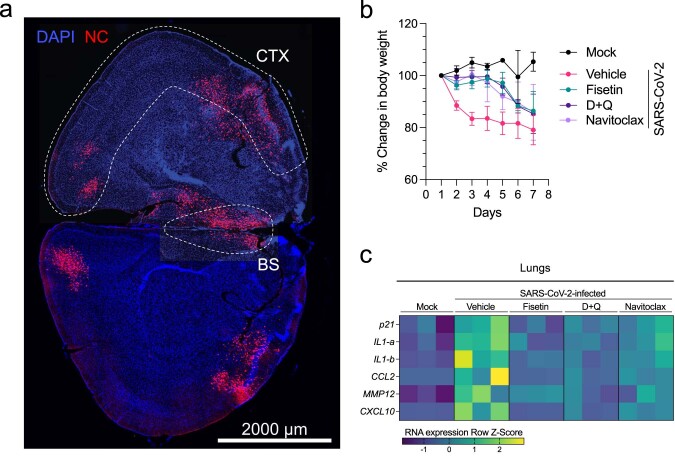
Senolytics delay weight loss and reduce lung senescence in SARS-CoV-2-infected K18-hACE2 mice. **(a)** Representative immunofluorescent images of viral nucleocapsid (NC) antigen in whole brain coronal sections of brains from SARS-CoV-2-infected K18-hACE2 transgenic mice (5 days post infection). CTX: Cerebral cortex; BS: Brainstem. **(b)** Percentage weight loss up to 7 days post infection. Uninfected mice (n = 3), and Delta SARS-CoV-2-infected mice treated with vehicle (n = 6), fisetin (n = 8), D + Q (n = 8), or navitoclax (n = 8). Error bars represent s.d. **(c)** Total RNA of individual lungs from mice uninfected or infected with the SARS-CoV-2 Delta variant and treated with various senolytic interventions was used to quantify the mRNA expression levels of the indicated senescence and SASP RNAs and was normalized to *Rplp0* mRNA. Each column in the heatmap represents an individual mouse lung analysed. Source data

To investigate the consequences of CNS SARS-CoV-2 infection and ensuing brain virus-induced senescence in a more physiologically complete system, we utilized transgenic mice expressing the human *ACE2* gene under the control of the keratin 18 promoter (K18-hACE2)^41^ and performed intranasal SARS-CoV-2 infections using the Delta variant, because it demonstrated the most significant virus-induced senescence in our human BO experiments. Notably, we found brain viral nucleocapsid antigen in cerebral cortex and brainstem regions (Extended Data Fig. 6a). Experimentally, 24 h post infection we initiated oral administration of the senolytic interventions navitoclax, fisetin and D + Q—drugs known to exert blood–brain barrier permeability^22,42^—with subsequent treatments every 2 days (Fig. 6a). As previously reported, SARS-CoV-2-infected K18-hACE2 transgenic mice underwent dramatically shortened lifespans following infection^41^, with a median survival of 5 days. Strikingly, treatment with D + Q or fisetin significantly improved the survival of K18-hACE2 mice compared with vehicle-treated controls, with extended median lifespans of 60% (Fig. 6b). Furthermore, while at 10 dpi all vehicle-treated control mice were already dead, at survival experimental endpoint (12 dpi) a percentage of senolytic-treated mice—22% (fisetin), 38% (D + Q) and 13% (navitoclax)—remained alive (Fig. 6b). This significantly improved survival following senolytic administration of infected mice concurrently delayed the rapid weight loss observed in the infected control group (Extended Data Fig. 6b). Throughout the first week of in vivo experiments, mice were clinically monitored and scored daily for behavioral and physical performance (Fig. 6c). Notably, senolytic interventions resulted in a profound reduction in COVID-related disease features, especially in the D + Q-treated group (Fig. 6c).

Given the positive survival and improved clinical performance outcomes induced by senolytic treatment, a phenomenon partially explained by reduction in lung senescence (Extended Data Fig. 6c) and pathology as previously shown^19,43^, we investigated whether the oral administration of senolytics would impact the histological architecture and proinflammatory makeup of brains from infected mice. To this end we first tested the impact of senolytics on brain viral RNA levels. In accordance with our brain organoid data (Fig. 5c), senolytic treatments of infected K18-hACE2 mice significantly lowered viral gene expression compared with infected vehicle-treated mice (Fig. 6d), further supporting a putative role for senescent cells in preferential sustenance of SARS-CoV-2 replication. We next tested whether senescent cell clearance would directly impact the transcription of SASP and senescence genes in the brain. mRNA expression analyses from brains of uninfected and infected mice indicated an overall increase in inflammatory SASP and p16 senescence markers in the brains of infected mice (Fig. 6e). Most importantly, all three senolytic interventions consistently normalized brain SASP and senescence gene expression of infected mice to levels comparable to those of uninfected brains (Fig. 6e).

Neuroinvasive viral infections can result in loss of dopaminergic neurons and ensuing PAIS such as parkinsonism^44^. Given the long-term neurological impact of COVID-19, including coordination and consciousness disorders^45^, we therefore tested the impact of SARS-CoV-2 infection on alteration of dopaminergic neuron survival within the brainstem, an important region of the brain known to regulate these behaviors. Strikingly, Delta variant infection induced a marked loss of dopaminergic neurons in the brainstem as measured by tyrosine hydroxylase (TH) immunolabeling (Fig. 6f,g), and this was accompanied by increased astrogliosis (Fig. 6f,h), a neurotoxic process common to multiple neurological disorders^46^. Importantly, recurrent senolytic treatments initiated 24 h following SARS-CoV-2 exposure partially prevented dopaminergic neuron loss and abrogated the onset of reactive astrogliosis (Fig. 6f–h).

## Discussion

Brain aging and related cognitive deficiency have been attributed to diverse molecular processes, including chronic inflammation and cellular senescence^47^. This has been studied in both normal murine aging^24^ and different age-related mouse models of neurodegeneration such as Parkinson’s disease^48^, tauopathies^23,49^, amyloid-beta neuropathology^22^ and neuropsychiatric disorders^50^. However, whether the endogenous age-related onset of cellular senescence impacts brain aging in human tissue systems has not been investigated. Neither have the putative consequences of neurotropic viral infections in accelerating the onset of cellular senescence in the brain been examined.

Our findings herein show that: (1) senescent cells accumulate in physiologically aged BOs of human origin and that long-term (4 weeks), intermittent senolytic treatment reduces inflammation and cellular senescence; (2) interventions unique to D + Q treatments induce antiaging and prolongevity gene expression changes in human BOs; (3) brains from COVID-19 patients undergo accelerated cellular senescence accumulation compared to age-matched controls; (4) SARS-CoV-2 and neurotropic viruses, including ZIKV and JEV, can infect human BOs to directly induce cellular senescence; (5) Delta (B.1.617.2) variant induces the strongest SARS-CoV-2-dependent induction of cellular senescence, where spatial transcriptomic sequencing of p16-positive cells identified a Delta-specific SASP signature; (6) short-term (5 days) senolytic treatments of SARS-CoV-2-infected organoids reduce viral gene expression and prevent the onset of senescent neurons of corticothalamic and GABAergic nature; and (7) senolytic treatment following SARS-CoV-2 intranasal infection of K18-hACE2 mice ameliorates COVID-19 neuropathology, including improvements in clinical score and survival, alleviation of reactive astrogliosis, increased survival of dopaminergic neurons and reduced viral, SASP and senescence gene expression in the brain of infected mice.

To evaluate the relationship between senescent cell accumulation and brain aging, we designed studies to eliminate senescent cells through pharmacologic approaches (D + Q, navitoclax and ABT-737) and hypothesized that senolytic interventions may have beneficial consequences in targeting brain aging. We found that physiologically aged human BOs accumulate senescent cells, mostly within astrocyte and mature neuron populations, and that senolytic treatment can be used as a proof-of-concept strategy to revert lamin B1 levels and alleviate differential SASP expression and senescent cell burden in human brain BO systems. In addition to senolytic activity, transcriptomic aging clocks identified D + Q as an intervention that achieved tissue rejuvenation, because 8-month-old human BOs displayed aging clocks comparable to those of D + Q-treated 9-month-old counterparts. Given that senescent cell clearance results in reversal of the aging process, these findings support an important role for senescent cells in driving human brain aging.

Further to normal brain aging, we tested the possibility of virus-induced senescence following BO neurotropic infections. We found that flaviviral JEV, ROCV and ZIKV infections—and multiple SARS-CoV-2 variant infections—led to a significant increase in BO cellular senescence. Importantly, following senolytic delivery, BOs showed a marked loss of SARS-CoV-2 viral RNA expression, suggestive of a role for senescent cells in preferential facilitation of viral entry and retention, consistent with data showing increased ACE2 expression in human senescent cells^51^. Furthermore, SARS-CoV-2 induces metabolic changes in infected and neighboring neurons^8^, a paracrine phenomenon reminiscent of the bystander effect characteristic of senescent cells^52^. Here, spatial transcriptomic sequencing cell deconvolution of p16 protein-expressing cell clusters identified two neuronal populations—corticothalamic and GABAergic—that become senescent and broadly develop a de novo SASP signature following Delta (B.1.617.2) infection. It will therefore be of interest to determine whether neuronal virus-induced senescence contributes to neuroinflammation and the long-term neurological impact of COVID-19.

In the brains of SARS-CoV-2-infected K18-hACE2 mice we found that senolytic treatment alleviates p16 and the levels of proinflammatory cytokines that may be due, in part, to removal of virus-induced senescence and ensuing SASP expression. However, secondary anti-inflammatory and/or antiviral effects of D + Q, fisetin or navitoclax—for instance, by direct inhibition of the observed astrogliosis—are also possible. Likewise, the crossing by navitoclax through the blood–brain barrier, particularly in young mice, is a matter of contention. As a result, the decline in senescence observed in the brains of infected mice may be an indirect consequence of senescent cell clearance in other organs, including the lungs. Following systematic monitoring of clinical performance in SARS-CoV-2-infected mice, we found that intermittent senolytic treatment significantly improved animal behavior and survival. This beneficial clinical effect of senolytics was associated with reduced brain inflammation and increased survival of dopaminergic neurons. Indeed, inflammatory cytokines as part of the SASP can impair brain plasticity^53^, suggesting that the beneficial effects of senolytic treatment on the COVID-19 neurological clinical picture may result from suppression of senescence-dependent inflammation and improved neuronal survival. This is consistent with preclinical studies demonstrating a beneficial effect of senescent cell clearance in reducing inflammatory/SASP gene expression in the brains of geriatric mice infected with a SARS-CoV-2-related mouse β-coronavirus^54^. Whether our in vivo effects of senolytics on COVID-19 neuropathology resulted solely from clearance of cellular senescence or also involved actions on dopaminergic neurons and other brain regions remains to be determined. Likewise, given the nature of the in vivo treatments, it is conceivable that the observed results on brain pathology are a consequence of a systemic impact caused by improved lung function. It is, however, crucial to emphasize the important contribution of our human BO experiments because they eliminate the potential confounding effects present in infections affecting multiple organs in our in vivo experiments. Indeed, the data from BOs provide compelling evidence of a distinct senescence phenotype resulting from direct infection by SARS-CoV-2, as well as from other neurotropic viruses such as ZIKV. Moreover, targeted treatment of infected organoids with senolytics shows a significant alleviation of virus-induced senescence and the associated secretome, indicating the direct efficacy of our interventions in cell types of the central nervous system. This pharmacological outcome remains independent of any potential secondary senescence that could have originated from other infected organs in a model conducted in vivo. In this study we have provided important evidence that paves the way for future clinical studies that will test the hypothesis that senolytic therapies can suppress long-COVID neuropathology and other long-term disorders caused by acute neurotropic viral infections.

## Methods

### Ethics and biological safety

All animal experiments were performed according to guidelines promoting the wellbeing of animals used for scientific research from The University of Queensland (UQ), and according to the Australian code for the care and use of animals for scientific purposes. The use of animals was approved by the UQ Animal Ethics Committee under project no. 2021/AE001119. Mice were housed within the BSL-3 facility using the IsoCage N-Biocontainment System (Tecniplast), where each cage was supplied with a high-efficiency, particulate-absorbing filter preventing viral contamination between cages. This IsoCage system also provides individual ventilation to the cages, maintaining humidity at <65–70% and temperature 20–23 °C. Mice were kept under a 12/12 h light/dark cycle with food and water provided ad libitum.

Pathogenic SARS-CoV-2 variants and encephalitic flaviviruses were handled under certified biosafety level-3 (BSL-3) conditions at the School of Chemistry and Molecular Biosciences (SCMB), Australian Institute for Bioengineering and Nanotechnology and Institute for Molecular Bioscience at UQ. All approved researchers used disposable Tychem 2000 coveralls (Dupont, no. TC198T YL) at all times and also used either powered air-purifying respirators (PAPR, SR500 Fan Unit) or Versaflo‐powered air‐purifying respirators (3M, no. 902‐03‐99) as respiratory protection. All pathogenic materials were handled in a class II biosafety cabinet within the BSL-3 facility. For downstream analysis, all samples containing infectious viruses were appropriately inactivated in accordance with the BSL-3 manual. Liquid and solid waste were steam sterilized by autoclave. This study was approved by the Institutional Biosafety Committee from UQ under approval nos. IBC/485B/SCMB/2021 and IBC/447B/SCMB/2021. Differentiation of hPSCs into organoids and their subsequent use was approved by the UQ Institutional Research Ethics Committee under approval no. 2019000159. The WA09 PSC cell line was obtained before this study following receipt of informed consent and approval by the NIH hESC Registry (no. NIHhESC-10-0062). Analysis of human brain sections was performed with the approval of the Ethics Committee of the University of Freiburg (no. 10008/09). Consent for autopsy was provided by the individuals’ next of kin or healthcare proxy according to German law (participant compensation was not applicable). The study was performed in agreement with the principles expressed in the Declaration of Helsinki, 2013.

### Generation and culture of PSC-derived human BOs

Organoid generation was carried out as previously described^55^, with some modifications. Human WA09 hPSCs were obtained, contamination free, from WiCell, with verified normal karyotype and were routinely tested and confirmed negative for mycoplasma (MycoAlert, Lonza). hPSCs were maintained in mTeSR medium (STEMCELL Technologies, no. 85850) on matrigel-coated plates (Corning, no. 354234). On day 0 of organoid differentiation, PSCs were dissociated with Accutase (Life Technologies, no. 00-4555-56) and seeded at a density of 15,000 cells per well on a 96-well, low-attachment U-bottom plate (Sigma, no. CLS7007) in mTeSR plus 10 μM ROCK inhibitor (VWR, no. 688000-5). The 96-well plate was then spun at 330*g* for 5 min to aggregate the cells and create spheroids. The spheroids were fed every day for 5 days in medium containing DMEM/F12 (Invitrogen, no. 11330-032), knockout serum (Invitrogen, no. 11320-033), 1:100 GlutaMax, 1:200 MEM-NEAA supplemented with dual SMAD inhibitors, 2 μM dorsomorphin (StemMACS, no. 130-104-466) and 2 μM A-83-01 (Lonza, no. 9094360). On day 6, half of the medium was changed to induction medium containing DMEM/F12, 1:200 MEM-NEAA, 1:100 GlutaMax, 1:100 N2 supplement (Invitrogen, no. 17502048) and 1 μg ml^–1^ heparin (Sigma, no. H3149) supplemented with 1 μM CHIR 99021 (Lonza, no. 2520691) and 1 μM SB-431542 (Sigma, no. S4317). From day 7, complete medium change was carried out with induction medium followed by daily medium change in induction medium for the next 4 days. On day 11 of the protocol, spheroids were transferred to 10 μl droplets of Matrigel on a sheet of Parafilm with 2 mm dimples. These droplets were allowed to gel at 37 °C for 25 min and were subsequently removed from the Parafilm and transferred to, and maintained in, low-attachment 24-well plates (Sigma, no. CLS3473) containing induction medium for the following 5 days. From day 16 the medium was then changed to organoid medium containing a 1:1 mixture of neurobasal medium (Invitrogen, no. 21103049) and DMEM/F12 medium supplemented with 1:200 MEM-NEAA, 1:100 GlutaMax, 1:100 N2 supplement, 1:50 B27 supplement (Invitrogen, no. 12587010), 1% penicillin/streptomycin (Sigma, no. P0781), 50 μM 2-mercaptoethanol and 0.25% insulin solution (Sigma, no. I9278). Medium was changed every other day with organoid medium. BOs were maintained in organoid medium until the end of experiments, as indicated. Microglia-containing organoid generation was carried out as previously described^56^ and these BOs were matured for 3 months before SARS-CoV-2 exposure at MOI = 1.

### Human tissue preparation

Frontal cortex tissue from patients that had tested positive for SARS-CoV-2 and died from severe COVID-19 was obtained at the University Medical Center Freiburg, Germany. Tissue was formalin fixed and embedded in paraffin using a Tissue Processing Center (Leica, no. ASP300). Sections (3 µm thick) were cut and mounted on Superfrost objective slides (Langenbrinck).

### Cell lines

RNA Vero E6 cells (African green monkey kidney cell clones) and TMPRSS2-expressing Vero E6 cell lines were maintained in DMEM (Gibco) at 37 °C with 5% CO_2_. In addition, as previously described, the TMPRSS2-expressing Vero E6 cell line was supplemented with 30 μg ml^–1^ puromycin^57^. C6/36 cells, derived from the salivary gland of the mosquito *A. albopictus*, were grown at 28 °C in RPMI medium (Gibco). All cell line media were supplemented with 10% heat-inactivated fetal calf serum (Bovogen), penicillin (100 U ml^–1^) and streptomycin (100 μg ml^–1^. C6/36 medium was also supplemented with 1% GlutaMax (200 mM, Gibco) and 20 mM HEPES (Gibco). All cell lines used in this study were tested for mycoplasma by first culturing cells for 3–5 days in antibiotic-free medium and then subjecting them to mycoplasma testing using the MycoAlert PLUS Mycoplasma Detection Kit (Lonza).

### Viral isolates

Seven SARS-CoV-2 variants were used in this study: (1) ancestral or Wuhan strain: an early Australian isolate, hCoV-19/Australia/QLD02/2020, sampled on 30 January 2020 (Global Initiative on Sharing All Influenza Data (GISAID) Accession ID: EPI_ISL_407896); (2) Alpha (B.1.1.7), named hCoV-19/Australia/QLD1517/2021 and collected on 6 January 2021 (GISAID accession ID: EPI_ISL_944644); (3) Beta (B.1.351), hCoV-19/Australia/QLD1520/2020, collected on 29 December 2020 (GISAID accession ID: EPI_ISL_968081); (4) Delta (B.1.617), hCoV-19/Australia/QLD1893C/2021 collected on 5 April 2021 (GISAID accession ID: EPI_ISL_2433928); (5) Gamma (P.1), hCoV-19/Australia/NSW4318/2021 sampled on 1 March 2021 (GISAID accession ID: EPI_ISL_1121976); (6) Lambda (C.37), hCoV-19/Australia/NSW4431/2021 collected on 3 April 2021 (GISAID accession ID: EPI_ISL_1494722); and (7) Omicron (BA.1), hCoV-19/Australia/NSW-RPAH-1933/2021 collected on 27 November 2021 (GISAID accession ID: EPI_ISL_6814922). All viral isolates obtained were passaged twice, except for Gamma and Lambda variants, which were passaged three times. Viral stocks were generated on TMPRSS2-expressing Vero E6 cells to ensure no Spike furin cleavage site loss. To authenticate SARS-CoV-2 isolates used in the study, viral RNA was extracted from stocks using TRIzol LS reagent (Thermo Fisher Scientific) and complementary DNA was prepared with a Protoscript II first-strand cDNA synthesis kit according to the manufacturer’s protocol (New England Biolabs). The full-length Spike glycoprotein was subsequently amplified with Prime Star GXL DNA polymerase (Takara Bio) and the following primers: CoV-SF GATAAAGGAGTTGCACCAGGTACAGCTGTTTTAAG, CoV-SR GTCGTCGTCGGTTCATCATAAATTGGTTCC, under conditions previously described^57^. For encephalitic flaviviruses, virulent strains of ZIKV (Natal (GenBank: KU527068.1)), JEV (Nakayama strain (GenBank: EF571853.1)) and ROCV (GenBank: AY632542.4) were propagated on C6/36 to generate viral stock for all experiments. Viral titers were determined by immunoplaque assay^58^.

### RNA isolation

RNA from BOs and mouse tissue was extracted with the RNeasy Mini Kit (Qiagen) for mRNA detection, according to the manufacturer’s instructions. Mouse tissue was homogenized with a TissueLyser II (Qiagen) at 30 Hz for 60 s. RNA integrity of BOs and mouse tissue was evaluated by analysis on a 2100 Bioanalyzer RNA 6000 Pico Chip kit (Agilent) using RNA integrity number. RNA samples with RNA integrity number >7 were considered to be of sufficiently high quality for real-time quantitative PCR, and for transcriptomic library construction and RNA-seq, according to the manufacturer’s instructions.

### Real-time quantitative PCR

Total RNA (1 μg) was reverse transcribed using an iScript cDNA Synthesis Kit (Bio-Rad). A volume corresponding to 5 ng of initial RNA was utilized for each real-time PCR reaction using PowerUp SYBR Green Master Mix (Applied Biosystems) on a CFX Opus Real-Time PCR detection system (data collection was performed via Bio-Rad’s CFX Maestro Software, v.2.3). Ribosomal protein P0 (RPLP0) was used as control transcript for normalization. Primer sequences (5'–3' orientation) are listed in Supplementary Table 1.

### BOs

Brain organoids on low-adhesion plates were infected overnight (14 h) with the indicated flaviviruses and SARS-CoV-2 variants at MOI = 0.1 and 1.0, respectively; BOs were then washed three times with lipopolysaccharide-free PBS, with the addition of maintenance medium, and maintained for 5 dpi.

### Senolytic treatments in vitro

For infection experiments, 5 days following viral exposure, BOs were treated with a single dose of either navitoclax (2.5 μM), ABT-737 (10 μM) or D + Q (D, 10 μM; Q, 25 μM) and monitored for 5 days following treatment. In regard to senolytic interventions on physiologically aged 8-month-old organoids, BOs were treated with a weekly dose of either navitoclax (2.5 μM), ABT-737 (10 μM) or D + Q (D, 10 μM; Q, 25 μM) for 4 weeks and subsequently collected for downstream analysis.

### SARS-CoV-2-driven COVID-19 animal experiments

In vivo experiments were performed using 6-week-old K18-hACE2 transgenic female mice obtained from the Animal Resources Centre (Australia). For animal infections, SARS-CoV-2 was delivered intranasally—20 μl of the Delta variant at 5 × 10^3^ focus-forming units per mouse—on anesthetized mice (100 mg kg^–1^ ketamine and 10 mg kg^–1^ xylazine). Control animals were mock infected with the same volume of RPMI additive-free medium. One day following infection, K18-hACE2 mice were distributed among three treatment groups (*n* = 16 each) and one solvent-only control group (*n* = 16). From 1 dpi, animals were treated by oral gavage with either navitoclax (100 mg kg^–1^), D + Q (D, 5 mg kg^–1^; Q, 50 mg kg^–1^) or fisetin (100 mg kg^–1^) dissolved in 5% DMSO and 95% corn oil every other day. For tissue characterization (*n* = 8 for each infected group), at 6 dpi animals were euthanized and brain specimens collected for RNA expression analysis and histopathological assessment. For clinical and survival evaluation, mice were monitored daily for up to 12 dpi. Clinical scoring included the following: no detectable disease (0); hindlimb weakness, away from littermates or ruffled fur (0.5–1.0); partial hindlimb paralysis, limping, hunched or reluctance to move (1.5–2.0); and complete paralysis of hindlimb, severely restricted mobility, severe distress or death (2.5–3.0).

### Organoid sectioning and histology

Brain organoids were fixed in 4% paraformaldehyde for 1 at room temperature (RT) and washed with PBS three times for 10 min each at RT before being allowed to sink in 30% sucrose at 4 °C overnight, and were then embedded in optimal cutting temperature (OCT; Agar Scientific, no. AGR1180) and cryosectioned at 14 μm with a Thermo Scientific NX70 Cryostat. Tissue sections were used for both immunofluorescence and SA-β-gal assay. For immunofluorescence, sections were blocked and permeabilized in 0.1% Triton X-100 and 3% bovine serum albumin in PBS. Sections were incubated with primary antibodies overnight at 4 °C, washed and incubated with secondary antibodies for 40 min at RT. DAPI (0.5 μg ml^–1^; Sigma, no. D9564) was added to secondary antibodies to mark nuclei. Secondary antibodies labeled with Alexafluor 488, 568 or 647 (Invitrogen) were used for detection. SA-β-gal activity at pH 6.0 as a senescence marker in fresh or cryopreserved human samples was assessed as previously described^59^.

### Nanostring spatial transcriptomics

OCT-embedded organoids were freshly sectioned and prepared according to the GeoMX Human Whole Transcriptome Atlas Assay slide preparation for RNA profiling (NanoString). Three organoids were used per condition for ROI selection. Fastq files were uploaded to the GeoMX DSP system, where raw and Q3-normalized counts of all targets were aligned with ROIs. The 0.75 quantile-scaled data were used as input. The DESeq2 v.1.30.1 R package^60^ was used to identify differently expressed genes in ROI cell subsets. DESeq2 was performed among pairwise comparisons of interest and genes were corrected using Benjamini–Hochberg correction, with only genes with corrected *P* < 0.05 retained. Cell abundance was estimated using the SpatialDecon v.1.10.0 R library, which performs mixture deconvolution using constrained log-normal regression and infers cell distributions based on pre-existing single-cell sequencing cell type annotations. Gene expression patterns of GeoMx data were deconvolved based on a training matrix of single-cell sequencing data from the Allen Human Brain Atlas. Projected proportions of different cell types were inferred, and are explained by the overall expression patterns and cell number of each of the spatial trancriptomic regions of interest.

### Whole-organoid RNA-seq

Before mRNA sequencing, ribosomal RNA from BO RNA was depleted using the Ribo-Zero rRNA Removal Kit (Illumina). Transcripts were sequenced at Novogene using TruSeq stranded total RNA library preparation and the Illumina NovaSeq 150-base pair, paired-end lane. FastQC was used to check the quality of raw sequences before analysis to confirm data integrity. Trimmed reads were mapped to human genome assembly hg38 using Hisat2 v.2.0.5. To ensure high quality of the count table, the raw count table generated by featureCounts v.1.5.0-p3 was filtered for subsequent analysis. Differential gene expression analysis was performed using Bioconductor DESeq2 R packages. The resulting *P* values were adjusted using the Benjamini–Hochberg approach for control of false discovery rate. Genes with adjusted *P* < 0.05 found by DESeq2 were assigned as differentially expressed.

### Association with gene expression signatures of aging and longevity

To assess the effect of senolytics on the transcriptomic age of BO samples, we applied a brain multispecies (mouse, rat, human) transcriptomic clock based on signatures of aging identified in ref. ^30^. Missing values were omitted with the precalculated average values from the clock. Association of gene expression log-fold change (FC) induced by senolytics in aged BO with previously established transcriptomic signatures of aging and established lifespan-extending interventions was examined, as described in ref. ^30^. Signatures of aging utilized included multispecies brain signature as well as multitissue aging signatures of mouse, rat and human. Signatures of lifespan-extending interventions included genes differentially expressed in mouse tissues in response to individual interventions including CR, rapamycin (Rapamycin) and mutations associated with growth hormone deficiency (GH deficiency), along with common patterns of lifespan-extending interventions (Common) and endothelial cells (ECs) associated with the intervention effect on mouse maximum (Max lifespan) and median lifespan (Median lifespan).

For identification of enriched functions affected by senolytics in aged BO, we performed functional gene set enrichment analysis (GSEA)^61^ on a preranked list of genes based on log_10_(*P*) corrected by the sign of regulation, calculated as

$$-({Pv})\times \mathrm{sgn}({I{\mathrm{FC}}}),$$

where *Pv* and *l*FC are *P* and logFC, respectively, of a particular gene obtained from edgeR output, and sgn is the signum function (equal to 1, –1 or 0 if the value is positive, negative or equal to 0, respectively). HALLMARK ontology from the Molecular Signature Database was used as gene sets for GSEA. The GSEA algorithm was performed separately for each senolytic via the fgsea package in R, with 5,000 permutations. A *q* value cutoff of 0.1 was used for selection of statistically significant functions.

Similar analysis was performed for gene expression signatures of aging and lifespan-extending interventions. Pairwise Spearman correlation was calculated for individual signatures of senolytics, aging and lifespan-extending interventions based on estimated normalized enrichment score (NES) (Fig. 2g). A heatmap colored by NES was built for manually chosen statistically significant functions (adjusted *P* < 0.1) (Extended Data Fig. 1a). A complete list of functions enriched by genes perturbed by senolytics is included in Source data.

### Imaging and analysis

Immunofluorescence images were acquired using either a Zeiss LSM 900 Fast Airyscan 2 super-resolution microscope or a Zeiss AxioScan Z1 Fluorescent Imager. For organoid staining, the number of positive cells per organoid for senescence, cell type and viral markers tested was analyzed using the imaging software CellProfiler (v.4.2.1) and Fiji (v.2.1.0/1.53c), with the same pipeline for each sample in the same experiment. Custom Matlab R2018b (9.5.0.944444) scripts were developed to streamline high-throughput imaging data. The CellProfiler pipeline for the quantification of SA-β-gal-positive cells is available in Supplementary Code 1.

### Antibodies

The following were used: anti-p16 (Cell Signalling, 1:400, no. 80772); anti-p21 (R&D Systems, 1:400, no. AF1047); anti-NeuN (Millipore, 1:1,000, no. ABN78); anti-GFAP (Agilent, 1:2,000, no. Z0334); anti-GFAP (Invitrogen, 1:1,000, no. 13-0300); anti-Sox2 (Cell Signalling, 1:1,000, no. 23064); anti-Sox2 (Cell Signalling, 1:1,000, no. 4900); anti-Sox10 (abcam, 1:500, no. ab229331); anti-Iba1 (Wako, 1:1,000, no. 019-19741); anti-SARS-CoV-2 Nucleocapsid C2, 1:1,000 (ref. ^62^); anti-SARS-CoV-2 Spike protein, 1:1,000 (ref. ^63^); anti-γH2AX (Millipore, 1:1,000, no. 05-636); anti-TH (Invitrogen, 1:1,000, no. PA5-85167); anti-lamin B1 (abcam, 1:5,000, no. ab16048); anti-chicken IgG (Jackson ImmunoResearch, 1:500, no. 703-545-155); anti-rabbit IgG (Invitrogen, 1:400, no. A10042); anti-rabbit IgG (Invitrogen, 1:400, no. A21245); anti-mouse IgG (Invitrogen, 1:400, no. A11029); anti-mouse IgG (Invitrogen, 1:400, no. A21235); and anti-human IgG (Invitrogen, 1:400, no. A21445).

### Statistics and reproducibility

All experiments were performed at least two times, except for RNA-seq, mouse experiments and human postmortem analysis. For in vivo experiments, 8–16 mice were analyzed per condition. All bar graph results are shown as mean ± s.e.m. or s.d. as indicated. No statistical methods were used to predetermine sample size, but our sample sizes are similar to those reported in previous publications^19,43^. Data distribution was assumed to be normal, but this was not formally tested. No data were excluded from analyses. Data collection and analysis were not performed blind to the conditions of the experiments, with the exception of mouse treatment experiments where the study investigators (E.A.A. and A.A.A.) were blinded to treatment groups by the use of color-coded drug vials generated by an independent investigator (J.A.). No randomization method was used to allocate animals or BOs to experimental groups. *P* values were calculated by the indicated statistical tests using either R (v.3.6.0), Microsoft Excel (v.16.77) or GraphPad Prism (v.9.4.0) software. In figure legends *n* indicates the number of independent experiments or biological replicates.

### Reporting summary

Further information on research design is available in the Nature Portfolio Reporting Summary linked to this article.

### Supplementary information

Reporting Summary

Supplementary Code 1. Pipeline for analysis of SA-β-gal-positive cells.

Supplementary Table 1. Sequences of oligonucleotides used in the present study.

### Source data

Source Data Fig. 1. Statistical source data for Fig. 1.

Source Data Fig. 2. Statistical source data for Fig. 2.

Source Data Fig. 3. Statistical source data for Fig. 3.

Source Data Fig. 4. Statistical source data for Fig. 4.

Source Data Fig. 5. Statistical source data for Fig. 5.

Source Data Fig. 6. Statistical source data for Fig. 6.

Source Data Extended Data Fig. 1. Statistical source data for Extended Data Fig. 1.

Source Data Extended Data Fig. 2. Statistical source data for Extended Data Fig. 2.

Source Data Extended Data Fig. 3. Statistical source data for Extended Data Fig. 3.

Source Data Extended Data Fig. 4. Statistical source data for Extended Data Fig. 4.

Source Data Extended Data Fig. 5. Statistical source data for Extended Data Fig. 5.

Source Data Extended Data Fig. 6. Statistical source data for Extended Data Fig. 6.

## Data Availability

RNA-seq raw data have been deposited in the European Nucleotide Archive with primary accession code PRJEB58180. RNA-seq files accessed from Mavrikaki et al.^18^ are available through Gene Expression Omnibus (accession no. GSE188847). The sequences of encephalitic flaviviruses used in this study are available in GenBank under accession nos. KU527068.1 (ZIKV), EF571853.1 (JEV) and AY632542 .4 (ROCV). For the SARS-CoV-2 variants used in this study, sequences are available in GISAID under accession nos. EPI_ISL_407896 (Wuhan strain), EPI_ISL_944644 (Alpha, B.1.1.7), EPI_ISL_968081 (Beta, B.1.351), EPI_ISL_2433928 (Delta, B.1.617), EPI_ISL_1121976 (Gamma, P.1), EPI_ISL_1494722 (Lambda, C.37) and EPI_ISL_6814922 (Omicron, BA.1). Source data are provided with this paper.

## Code availability

Code used for SA-β-gal image quantifications is available in the Supplementary material. Additional custom code used for processing of imaging data is available from the corresponding author on reasonable request.
