# Supplementary material for: Senolytic therapy alleviates physiological human brain aging and COVID-19 neuropathology
Source: Nat Aging. 2023 Nov 13;3(12):1561–75. doi: 10.1038/s43587-023-00519-6 (PMC10724067; doi:10.1038/s43587-023-00519-6)
Supplement: Supplementary file 1 — Reporting Summary [file 43587_2023_519_MOESM1_ESM.pdf]

Corresponding author(s): Julio Aguado, PhD

Last updated by author(s): Sep 18, 2023

Reporting Summary

Nature Portfolio wishes to improve the reproducibility of the work that we publish. This form provides structure for consistency and transparency in reporting. For further information on Nature Portfolio policies, see our Editorial Policies and the Editorial Policy Checklist.

Statistics

For all statistical analyses, confirm that the following items are present in the figure legend, table legend, main text, or Methods section.

n/a	Confirmed	
☐	☒	The exact sample size (*n*) for each experimental group/condition, given as a discrete number and unit of measurement
☐	☒	A statement on whether measurements were taken from distinct samples or whether the same sample was measured repeatedly
☐	☒	The statistical test(s) used AND whether they are one- or two-sided<br>*Only common tests should be described solely by name; describe more complex techniques in the Methods section.*
☐	☒	A description of all covariates tested
☐	☒	A description of any assumptions or corrections, such as tests of normality and adjustment for multiple comparisons
☐	☒	A full description of the statistical parameters including central tendency (e.g. means) or other basic estimates (e.g. regression coefficient) AND variation (e.g. standard deviation) or associated estimates of uncertainty (e.g. confidence intervals)
☐	☒	For null hypothesis testing, the test statistic (e.g. *F*, *t*, *r*) with confidence intervals, effect sizes, degrees of freedom and *P* value noted<br>*Give P values as exact values whenever suitable.*
☒	☐	For Bayesian analysis, information on the choice of priors and Markov chain Monte Carlo settings
☒	☐	For hierarchical and complex designs, identification of the appropriate level for tests and full reporting of outcomes
☒	☐	Estimates of effect sizes (e.g. Cohen's *d*, Pearson's *r*), indicating how they were calculated

Our web collection on statistics for biologists contains articles on many of the points above.

Software and code

Policy information about availability of computer code

Data collection	qPCR data collection were performed by CFX Maestro Software (BioRad, version 2.3)<br>Immunofluorescence images were acquired using a Zeiss LSM 900 Fast Airyscan 2 super-resolution microscope or a Zeiss AxioScan Z1 Fluorescent Imager; and imaging data collection was performed by ZEN lite (ZEISS)
Data analysis	Data visualization and analysis : GraphPad Prism (version 9.4.0)<br>Image analysis: Fiji (Version: 2.1.0/1.53c)<br>Data visualization and analysis: Microsoft Office Excel (Version 16.77)<br>Image analysis: MATLAB_R2018b (9.5.0.944444)<br>Image analysis: CellProfiler 4.2.1<br>Transcriptomic analysis: R version 3.6.0

For manuscripts utilizing custom algorithms or software that are central to the research but not yet described in published literature, software must be made available to editors and reviewers. We strongly encourage code deposition in a community repository (e.g. GitHub). See the Nature Portfolio guidelines for submitting code & software for further information.

Data

Policy information about availability of data

All manuscripts must include a data availability statement. This statement should provide the following information, where applicable:

- Accession codes, unique identifiers, or web links for publicly available datasets
- A description of any restrictions on data availability
- For clinical datasets or third party data, please ensure that the statement adheres to our policy

RNA-seq raw data have been deposited in the European Nucleotide Archive with the primary accession code PRJEB58180. RNA-seq files from Mavrikaki et al. are available through the Gene Expression Omnibus accession number GSE188847. The sequences of encephalitic flaviviruses used in this study are available in GenBank under accession numbers KU527068.1 (ZIKV), EF571853.1 (JEV) and AY632542.4 (ROCV). For the SARS-CoV-2 variants used in this study, sequences are available in the Global Initiative on Sharing All Influenza Data (GISAID) under accession numbers EPI_ISL_407896 (Wuhan strain), EPI_ISL_944644 (Alpha: B.1.1.7), EPI_ISL_968081 (Beta: B.1.351), EPI_ISL_2433928 (Delta: B.1.617), ID EPI_ISL_1121976 (Gamma: P.1), EPI_ISL_1494722 (Lambda: C.37) and EPI_ISL_6814922 (Omicron: BA.1). Source data files are provided with this paper.

Human research participants

Policy information about studies involving human research participants and Sex and Gender in Research.

Reporting on sex and gender	Age data for the anonymized COVID-19 tissue specimens is consisted exclusively of individuals with an age of approximately 75 years. On that basis, control age-matched non-COVID tissue samples were selected as the reference. No information on gender is available.
Population characteristics	Data on population characteristics is unavailable.
Recruitment	Human Formaldehyde-fixed paraffin-embedded brain sections from COVID-19 patients (n = 7), and age-matched control patients (n = 8) were obtained postmortem. COVID-19 patients were autopsied upon order issued by the Hamburg public health authorities in accordance with section 25(4) of the German Infection Protection Act.
Ethics oversight	The analvsis of human brain sections was performed with the approval of the Institutional Review Board (University of Freiburg: 10008/09). The study was performed in agreement with the principles expressed in the Declaration of Helsinki (2013).

Note that full information on the approval of the study protocol must also be provided in the manuscript.

Field-specific reporting

Please select the one below that is the best fit for your research. If you are not sure, read the appropriate sections before making your selection.

☒ Life sciences ☐ Behavioural & social sciences ☐ Ecological, evolutionary & environmental sciences

For a reference copy of the document with all sections, see nature.com/documents/nr-reporting-summary-flat.pdf

Life sciences study design

All studies must disclose on these points even when the disclosure is negative.

Sample size	No statistical methods were used to predetermine sample size but our sample sizes are similar to those reported in previous publications (eg. Lee et al, Nature 2021; Delval et al, Nature Aging 2023).
Data exclusions	No data were excluded from the analysis.
Replication	All of the experimental results were replicated as indicated in figure legends. Only biological replicates were plotted and used for statistical analyses. All experiments were performed at least two times except for RNA sequencing, mouse experiments and human postmortem analysis.
Randomization	For all experiments, animals and/or organoid cultures were randomly assigned to experimental groups.
Blinding	For molecular studies including imaging, sequencing and qPCR analyses, the experiments were not blinded, as all of the findings are supported by quantitative measurement.<br>During the phenotyping experiments of transgenic mice, the experiments were performed blinded and the conditions (± senolytic treatments) were only disclosed after data analyses.

Reporting for specific materials, systems and methods

We require information from authors about some types of materials, experimental systems and methods used in many studies. Here, indicate whether each material, system or method listed is relevant to your study. If you are not sure if a list item applies to your research, read the appropriate section before selecting a response.

Materials & experimental systems

n/a	Involved in the study
☐	☒ Antibodies
☐	☒ Eukaryotic cell lines
☒	☐ Palaeontology and archaeology
☐	☒ Animals and other organisms
☒	☐ Clinical data
☒	☐ Dual use research of concern

Methods

n/a	Involved in the study
☒	☐ ChIP-seq
☒	☐ Flow cytometry
☒	☐ MRI-based neuroimaging

Antibodies

Antibodies used	anti-p16 (Cell Signalling, 80772, 1:400); anti-p21 (R&D Systems, AF1047, 1:400); anti-NeuN (Millipore, ABN78, 1:1000); anti-GFAP (Agilent, Z0334, 1:2000); anti-GFAP (Invitrogen, 13-0300, 1:1000); anti-Sox2 (Cell Signalling, 23064, 1:1000); anti-Sox2 (Cell Signalling, 4900, 1:1000); anti-Sox10 (Abcam, ab229331, 1:500); anti-Iba1 (Wako, 019-19741, 1:1000); anti-SARS-CoV-2 Nucleocapsid C2 (from Isaacs et al. PMID: 35757714, 1:1000); anti-SARS-CoV-2 spike protein (from Modhiran et al. PMID: 37361875, 1:1000); anti-γH2AX (Millipore, 05-636, 1:1000); anti-Tyrosine Hydroxylase (Invitrogen, PA5-85167, 1:1000); anti-lamin B1 (Abcam, ab16048, 1:5000); anti-Chicken IgG (Jackson ImmunoResearch, 703-545-155, 1:500); anti-rabbit IgG (Invitrogen, A10042, 1:400); anti-rabbit IgG (Invitrogen, A21245, 1:400); anti-mouse IgG (Invitrogen, A11029, 1:400); anti-mouse IgG (Invitrogen, A21235, 1:400); anti-human IgG (Invitrogen, A21445, 1:400)
Validation	Antibodies were chosen because of their validation in prior publications.

Eukaryotic cell lines

Policy information about cell lines and Sex and Gender in Research

Cell line source(s)	Human embryonic stem cells (WA09) were obtained from WiCell.<br>Cercopithecus aethiops Vero E6 (Vero 76, clone E6, ATCC CRL-1568).<br>Aedes albopictus C6/36 (ATCC® CRL-1660).<br>Production of TMPRSS2 expressing VeroE6 cell lines (VeroE6-TMPRSS2) was established as indicated in Amarilla et al, Nature Communications 2021 (DOI: 10.1038/s41467-021-23779-5)
Authentication	Cell lines were authenticated by the provider, Master banks were established and cell lines were used within 10-15 passages after establishment. Cell lines were not authenticated by ourselves.
Mycoplasma contamination	All cells were routinely tested and confirmed mycoplasma-negative.
Commonly misidentified lines (See ICLAC register)	No commonly misidentified cell lines were used in this study.

Animals and other research organisms

Policy information about studies involving animals; ARRIVE guidelines recommended for reporting animal research, and Sex and Gender in Research

Laboratory animals	Six-week-old K18-hACE2 transgenic mice were purchased from the Animal Resources Centre (Australia). Animals were housed in groups of six and fed standard diets ad libitum. Virus inoculations were performed under anesthesia and all efforts were made to minimize animal suffering.
Wild animals	No wild animals were used for this study.
Reporting on sex	Female mice were used in the study, based on our and other investigators experience.
Field-collected samples	No field collections were used for this study.
Ethics oversight	Mouse experiments were approved by the University of Queensland Animal Ethics Committee (project 2021/AE001119) and conducted in accordance with the "Australian Code for the care and use of animals for scientific purposes" as defined by the National Health and Medical Research Council of Australia.

Note that full information on the approval of the study protocol must also be provided in the manuscript.
